# Addressing Glaucoma in Myopic Eyes: Diagnostic and Surgical Challenges

**DOI:** 10.3390/bioengineering10111260

**Published:** 2023-10-29

**Authors:** Kateki Vinod, Sarwat Salim

**Affiliations:** 1 Department of Ophthalmology, Icahn School of Medicine at Mount Sinai, New York Eye and Ear Infirmary of Mount Sinai, New York, NY 10003, USA; 2 Department of Ophthalmology, Tufts University School of Medicine, Boston, MA 02116, USA

**Keywords:** glaucoma, high myopia, myopia, optic disc, optical coherence tomography, perimetry, cataract surgery

## Abstract

Epidemiological and genetic studies provide strong evidence supporting an association between myopia and glaucoma. The accurate detection of glaucoma in myopic eyes, especially those with high myopia, remains clinically challenging due to characteristic morphologic features of the myopic optic nerve in addition to limitations of current optic nerve imaging modalities. Distinguishing glaucoma from myopia is further complicated by overlapping perimetric findings. Therefore, longitudinal follow-up is essential to differentiate progressive structural and functional abnormalities indicative of glaucoma from defects that may result from myopia alone. Highly myopic eyes are at increased risk of complications from traditional incisional glaucoma surgery and may benefit from newer microinvasive glaucoma surgeries in select cases.

## 1. Introduction

The global prevalence of myopia is rapidly increasing, with particularly high rates in Asia [1]. Approximately half the world population is predicted to have myopia by 2050 [2]. This increased prevalence raises additional concerns for vision-threatening ocular sequelae related to myopia, including myopic degeneration, retinal detachment, cataract, amblyopia, and glaucoma. A recent meta-analysis of 24 studies from 11 countries found a 20% increased risk of glaucoma for every one diopter increase in myopia [3].

Epidemiological studies provide robust data and support for myopia as a risk factor for open-angle glaucoma. The Blue Mountains Eye Study was a population-based study of Australian adults aged 49 or older in which glaucoma was observed more commonly in myopes (4.2% of eyes with spherical equivalent −1.0 to −3.0 diopters and 4.4% of eyes with spherical equivalent >−3.0 diopters) than those without myopia (1.5%) [4]. High myopia, commonly defined as a spherical equivalent >−6.0 diopters and/or an axial length > 26.5 mm, appears to confer an even greater risk of glaucoma. In the Beijing Eye Study, a population-based study of Chinese adults aged 40 or older, glaucoma was observed more frequently in eyes with marked (−6.0 to −8.0 diopters) and high myopia (>−8.0 diopters) versus those with moderate myopia (−3.0 to −6.0 diopters; *p* = 0.075), low myopia (−0.5 to −3.0 diopters; *p* = 0.001), emmetropia (−0.5 to +2.0 diopters; *p* < 0.001), and hyperopia (>+2.0 diopters; *p* = 0.005) [5]. In a 10-year follow-up study, low, moderate, and high myopia conferred a 3.2-, 4.2-, and 7.3-fold increased risk of glaucoma, respectively, as compared with emmetropia (*p* < 0.01) [6].

In the Beijing Eye Study, glaucoma was diagnosed based upon morphologic assessments of optic disc photographs. In general, myopic eyes often exhibit optic nerve features, optical coherence tomography of the retinal nerve fiber layer (OCT RNFL) and ganglion cell complex (GCC) thinning, and visual field defects that resemble, but are not always indicative of, glaucoma, thereby complicating its detection. This review focuses on recent advances and research on myopia as it relates to the pathophysiology, diagnosis, and surgical management of glaucoma.

## 2. Pathophysiology of Glaucoma in Myopic Eyes

The pathophysiology underlying the increased susceptibility of optic nerves in myopic eyes to glaucomatous damage is not fully understood. Optic nerves are thought to be more vulnerable to damage due to poor structural support for retinal nerve fibers in myopic eyes, particularly those with high myopia. Axial elongation likely results in thinning of the sclera and produces greater shearing forces across the stretched lamina cribrosa, leaving the optic nerve more susceptible to injury [7].

Intraocular pressure (IOP) is well established as the main and only modifiable risk factor for glaucoma and likely plays an important role in the pathophysiology of glaucoma in myopic eyes. Recent Mendelian randomization studies suggest that myopia and primary open-angle glaucoma (POAG) share a genetic basis, and that IOP is the primary mediator underlying their bidirectional causal relationship [8,9]. In the Singapore Epidemiology of Eye Diseases Study, eyes with myopia >−3.0 diopters and IOP ≥ 20 mm Hg were over four times more likely to have POAG versus eyes without myopia and normal IOP. Eyes with an axial length >25.5 mm and IOP ≥ 20 mm Hg were over 16 times more likely to have POAG versus eyes with an axial length <23.5 mm and normal IOP [10]. Axial length may therefore be a clinically relevant metric for risk stratification of myopic glaucoma suspects. Importantly, myopic eyes, especially those with high myopia, may develop glaucoma within the normal range of IOP due to underlying structural abnormalities in the sclera and lamina cribrosa [7]. 

Recent studies offer mixed results regarding a possible genetic correlation between POAG and myopia. Iglesias and associates analyzed data from the Australian & New Zealand Registry of Advanced Glaucoma study (N = 798 POAG patients and 1992 control patients) and the Rotterdam Study (N = 11,097) and did not find evidence supporting an association between polygenic risk scores of POAG and myopia [11]. Using data from the UK Biobank and Genetic Epidemiology Research on Adult Health and Aging (GERA) cohort (N = 154,018), Choquet and colleagues performed a genetic correlation analysis and found that patients with POAG had decreased mean spherical equivalents and a greater probability of having myopia or high myopia when compared with patients without POAG [8]. Additional studies in more diverse patient populations are needed to better elucidate a potential genetic basis for the increased glaucoma risk in myopic individuals that has been found in observational studies.

## 3. Diagnostic Structural and Functional Testing of Glaucoma in Myopic Eyes

Diagnosing glaucoma in a myopic eye presents numerous challenges, especially when the IOP is within the normal range. Inherent differences in corneal biomechanics may exist in eyes with high myopia as compared with emmetropic eyes and eyes with low myopia, and should be taken into consideration when interpreting measured IOP values. Importantly, rebound tonometry is likely influenced by corneal biomechanics to a greater extent than Goldmann applanation tonometry, and may underestimate IOP in eyes that develop glaucoma within the normal range of IOP [12]. Corneal biomechanical parameters include corneal hysteresis, a biomarker that quantifies the viscous dampening of the cornea, and corneal resistance factor, which is a largely IOP-independent measure of corneal resistance. A meta-analysis of eleven studies using the Ocular Response Analyzer (ORA) to evaluate corneal biomechanical indices observed significantly lower corneal hysteresis and corneal resistance factor among eyes with high (≥−6.0 diopters; N = 1027 eyes) versus low myopia (≤−3.0 diopters; N = 835 eyes), and found no difference in central corneal thickness [13]. These findings suggest that high myopia may be associated not only with decreased rigidity of the sclera, but also of the cornea. Clinicians may therefore consider obtaining corneal hysteresis measurements in highly myopic patients, particularly in those who exhibit glaucomatous progression despite seemingly “normal” IOP. Patients with myopia should also be asked about any history of corneal refractive surgery, which is known to further influence the accuracy of IOP measurements due to its effects on corneal biomechanical properties. 

**Figure 1:**
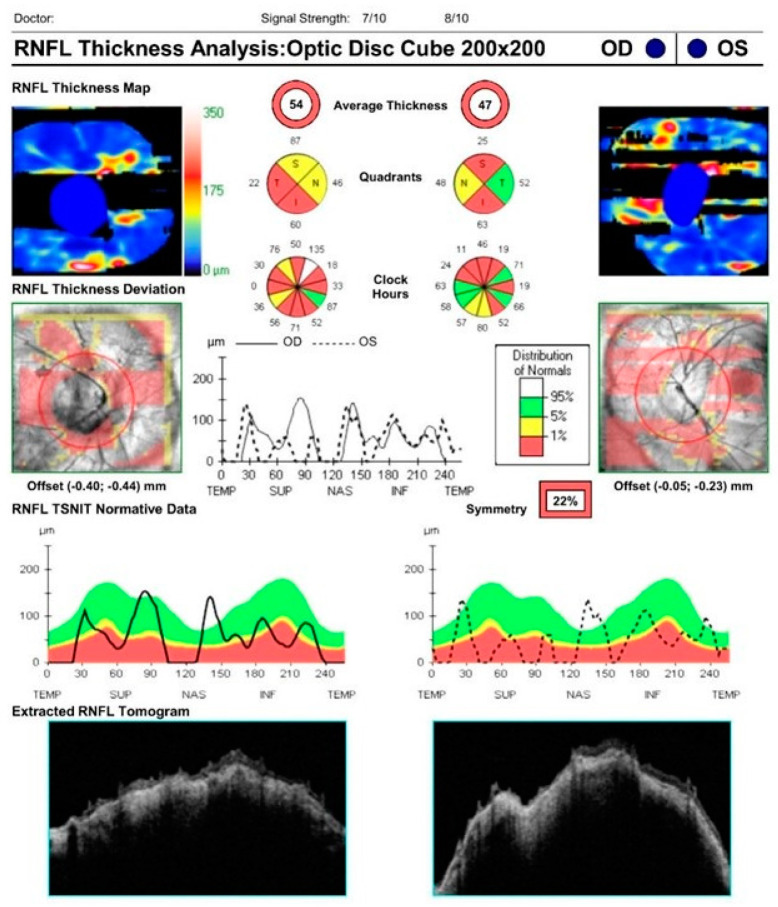
OCT RNFL demonstrating segmentation artifacts in a patient with high myopia, characteristic of “red disease”.

Clinically, myopic optic nerves may be difficult to differentiate from glaucomatous optic nerves. Unique anatomical features of myopic optic discs can confound quantitative structural and functional assessments, which can lead to overtreatment or undertreatment in many cases. Myopic eyes often exhibit such characteristics as large-diameter optic discs, optic disc tilting or torsion, and beta-zone peripapillary atrophy. Such features can result in erroneous OCT measurements of the circumpapillary RNFL (Figure 1). In general, retinal thinning is common in myopic eyes and may be misinterpreted as glaucomatous thinning in the setting of a globally reduced circumpapillary RNFL measurement [14]. Kang and colleagues found that average RNFL thickness decreases by approximately 1.3 microns for every additional diopter of myopia [15]. Leung and associates observed an increasingly temporal orientation of the superotemporal and inferotemporal RNFL bundles with higher degrees of myopia, resulting in greater RNFL areas deemed “abnormal” in comparison with a normative database [16]. As a result of this deviation, temporal sectors may appear more robust while nasal sectors may appear thinner on circumpapillary RNFL measurements in myopic eyes, and RNFL probability maps may demonstrate arcuate artifacts mimicking glaucoma. Additionally, misalignment of the scan circle or the presence of extensive peripapillary atrophy crossing its margin may result in erroneous measurements. Retinal nerve fiber layer segmentation artifacts may also result from vitreopapillary traction or peripapillary retinoschisis. Therefore, clinicians must exercise vigilance in identifying “red disease”, whereby OCT RNFL may misclassify a healthy myopic nerve as “abnormal” based upon limited normative databases in commercially available OCT machines that exclude highly myopic eyes [14,17]. Specific attention must be paid not only to the global and sectoral circumpapillary RNFL thickness, but also to b-scans, circumpapillary RNFL plots, and RNFL and macular thickness maps [18]. 

While some studies suggest improved diagnostic accuracy of macular thickness over circumpapillary RNFL in identifying glaucoma in highly myopic eyes [19,20], others do not [21,22]. Like circumpapillary RNFL thickness, macular thickness is often globally reduced in high myopes. Segmentation errors also occur with macular imaging in myopic eyes. Hwang and colleagues observed a segmentation error rate of 9.7% in macular thickness scans from 538 eyes with and without glaucoma while excluding those with macular pathology, and these errors were reproducible in 46% of eyes. A higher degree of myopia was found to be significantly associated with the occurrence of segmentation errors (*p* < 0.001) [23].

Advances in OCT RNFL imaging have the potential to improve glaucoma detection in highly myopic eyes. Biswas and colleagues evaluated a new myopic normative OCT RNFL database in 180 eyes with high myopia (average spherical equivalent −8.0 ± 1.8 diopters), which showed improved specificity versus the standard normative database while maintaining sensitivity for detecting glaucoma [24]. Baek and associates incorporated topographic RNFL and ganglion cell–inner plexiform layer (GCIPL) parameters to develop a scoring system to identify glaucomatous damage in the setting of myopia. This combined approach topographic scoring system outperformed individual RNFL and GCIPL thickness parameters in both myopic and highly myopic eyes [25]. Kim and colleagues found that swept-source OCT wide-field maps displayed better accuracy for diagnosing glaucomatous defects in myopic eyes than spectral domain OCT, likely due to the former modality’s wider area of measurement [26].

**Figure 2:**
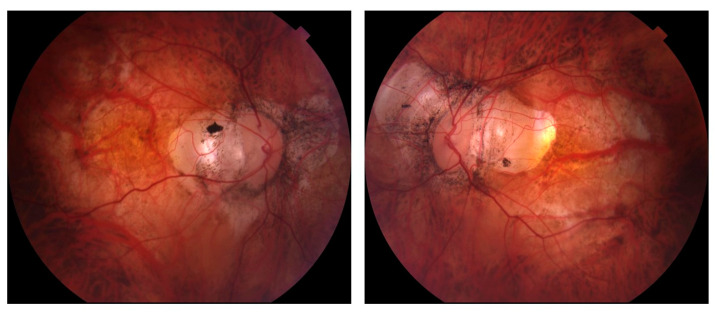
Optic disc photographs from a patient with high myopia demonstrating optic disc tilting and extensive peripapillary atrophy, making optic nerve assessment difficult.

While OCT RNFL and macular imaging have largely supplanted the use of stereoscopic disc photographs in the longitudinal assessment of glaucoma, the latter remain useful when following patients with myopia, particularly as OCT technology continues to evolve (Figure 2). Future directions to improve glaucoma detection in myopic and highly myopic eyes may include OCT angiography as well as artificial intelligence and deep learning strategies.

**Figure 3:**
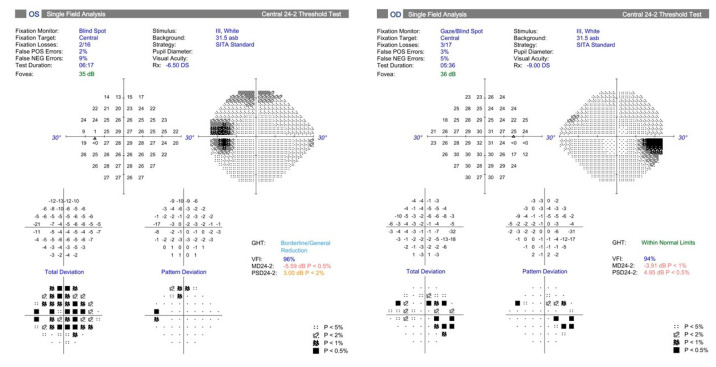
Visual fields from a patient with high myopia demonstrating enlarged blind spots.

Myopia is known to cause various visual field abnormalities that mimic glaucoma but typically do not progress over time as would glaucomatous defects in untreated eyes. A key component of glaucoma evaluation in myopic eyes, therefore, is longitudinal follow-up. Enlarged blind spots are common in high myopes (Figure 3), and tilted discs often produce superotemporal visual field defects [27]. A retrospective study of myopic glaucoma suspects aged 50 or younger found that myopia was associated with visual field defects resembling glaucoma, such as nasal steps, arcuate defects, and paracentral scotomas [28]. Doshi and colleagues demonstrated nonprogressive visual field defects mirroring glaucoma over a seven-year period in 16 myopic Chinese men (age range 25 to 66 years; mean 38.9), only approximately half of whom were using IOP-lowering medications. The authors postulated that visual field defects in myopic eyes may develop and progress during axial elongation earlier in life and later stabilize in adulthood in this specific subpopulation [29]. Han and associates performed a retrospective longitudinal study in Korea with a minimum follow-up of seven years and found that inferiorly tilted discs were more likely than temporally tilted discs to produce localized, single-hemifield visual field defects and to demonstrate visual field progression. Given that the large majority (92.5%) of visual field defects remained limited to the superior hemifield in eyes with inferiorly tilted optic discs over the study period, the authors proposed that there may be a finite extent to which progression can occur related to the focal vulnerability of a myopic optic nerve in the direction of optic disc tilt [30]. The authors also suggested that a new visual field defect in the opposite hemifield correlating to a previously healthy area of neuroretinal rim might be more suggestive of true glaucoma.

Interestingly, Lin and colleagues developed a classification system for visual field defects observed in highly myopic eyes without maculopathy. The authors used a database of 1893 visual fields from Chinese patients (mean age ± SD, 30.94 ± 9.75 years) enrolled in a prospective longitudinal registry study of high myopia, defined as a spherical equivalent ≥−6.00 diopters or axial length ≥26.5 mm. Visual fields were classified as being normal or as having high myopia-related abnormalities (i.e., enlarged blind spot, vertical step, partial peripheral rim, or nonspecific), glaucoma-related abnormalities (i.e., nasal step, partial or full arcuate, or paracentral), or combined defects (i.e., nasal step plus enlarged blind spot). Two trained graders twice analyzed a common set of 1000 visual fields representing all four categories. The intraobserver and interobserver agreements were found to be over 89%. The authors observed that 10.8% of highly myopic eyes showed glaucoma-like visual field defects, and the prevalence of such defects was associated with increased axial length [31]. Additional prospective studies are needed to better understand how morphologic features of myopic optic discs, including tilt, torsion, and peripapillary atrophy, predispose certain myopic eyes to the development and progression of visual field defects.

## 4. Surgical Considerations in Myopic Eyes

Numerous surgical options exist to treat glaucoma in myopic eyes, and each has its advantages and disadvantages (Table 1). An inevitable trade-off between safety and efficacy exists with regard to glaucoma surgery and is particularly relevant in highly myopic eyes. Decreased scleral rigidity in high myopes increases the likelihood of hypotony-related complications following glaucoma filtration surgery [32,33,34]. Hypotony maculopathy has been reported to occur with a delayed onset of 14 years following trabeculectomy in the absence of a bleb leak [35]. Given these risks, selective laser trabeculoplasty may be attempted earlier in myopic and highly myopic patients, prior to incisional surgery. Caution should be exercised when performing laser trabeculoplasty in myopic eyes with pigment dispersion syndrome or pigmentary glaucoma, as severe IOP elevation may occur postoperatively due to greater absorption of laser energy by the heavily pigmented trabecular meshwork characteristic of these conditions. The use of lower energy settings and/or a limited extent of treatment (i.e., 90 to 180 degrees, rather than 360 degrees) often mitigate this risk. If laser trabeculoplasty fails to control the IOP adequately or is not feasible, consideration may be given to microinvasive glaucoma surgery (MIGS) as a first surgical intervention in highly myopic eyes with early to moderate glaucoma. These newer procedures generally offer an improved safety profile over traditional incisional glaucoma surgery [36]. Furthermore, myopic eyes typically have wide open angles that facilitate trabecular and Schlemm’s canal-based procedures. However, subconjunctival MIGS, such as the XEN gel stent, may be associated with an increased incidence of hypotony and hypotony-related sequelae in highly myopic eyes, akin to those observed with trabeculectomy [37,38].

Angle-based MIGS may not offer sufficient IOP lowering in eyes with severe or rapidly progressive glaucomatous damage, and trabeculectomy may be required in certain cases. Intraoperative modifications to the standard trabeculectomy technique in highly myopic eyes may include an increased number of, and tighter, scleral flap sutures and cautious use of antimetabolites to mitigate the risk of hypotony in the early postoperative period. Alternatively, the use of valved glaucoma drainage implants or smaller-surface-area nonvalved implants may reduce the risk of hypotony and its sequelae. Additionally, scleral flap dissection during trabeculectomy and endplate placement during glaucoma drainage implant surgery must be performed carefully in the presence of thin sclera to avoid scleral perforation. Consideration should be given to performing a planned laser tube ligature release for nonvalved glaucoma drainage implants rather than allowing spontaneous tube ligature as the latter strategy may be associated with a higher likelihood of hypotony, anterior chamber shallowing, and other hypotony-related complications. During the early postoperative course following traditional incisional glaucoma surgery, surgeons should maintain a lower threshold to perform anterior chamber injection of cohesive viscoelastic in highly myopic eyes with hypotony to minimize complications. 

Trans-scleral cyclophotocoagulation is a reasonable alternative to incisional glaucoma surgery in highly myopic eyes. Cycloablation is generally associated with intraoperative pain, typically requiring the use of a peribulbar or retrobulbar block to achieve adequate anesthesia. However, peribulbar or retrobulbar blocks carry a risk of globe perforation in highly myopic eyes, and a sub-Tenon’s block may be a safer alternative. Intraoperative transillumination of the globe may be advisable during cyclophotocoagulation to confirm the location of the ciliary body, which may vary in highly myopic eyes [39]. Cyclophotocoagulation is a nontitrable procedure that is associated with numerous risks, including macular edema and hypotony. Surgeons should be aware of any history of previous cyclophotocoagulation when performing subsequent glaucoma surgery in patients with high myopia. Late hypotony with maculopathy has been described following uneventful viscocanalostomy in a highly myopic eye with a history of prior cycloablation [40].

Special considerations also exist when planning and performing phacoemulsification cataract surgery in myopic eyes with and without glaucoma. Patients should be counseled about the increased risk of intraoperative and postoperative complications as well as postoperative refractive surprises. In a study of 115 eyes with axial length ≥27 mm, Kora and colleagues found that erroneous axial length measurement was the main contributor to postoperative refractive errors [41]. Axial length may be overestimated by ultrasound biometry in highly myopic eyes with posterior staphylomas, resulting in hyperopic surprises. In patients with adequate fixation and without dense lens opacities, optical biometry is more likely to provide accurate axial length measurements than ultrasound [42]. Newer intraocular lens (IOL) calculation formulas, such as the fourth-generation Barrett Universal II and modified Wang–Koch adjustment to the third-generation Holladay I, may further reduce the risk of refractive surprises. Although published studies offer conflicting results, the majority suggest more optimal refractive outcomes when using the Haigis or Barrett Universal II formulas for IOL calculations in eyes with axial length <30 mm and the Barrett Universal II formula in eyes with axial length >30 mm [43]. Novel IOL calculation formulas that use artificial intelligence, including Kane and Hill-RBG3.0, appear to offer even greater accuracy in eyes with high myopia [44]. A history of prior refractive surgery in myopic eyes necessitates adjustment to standard formulas for intraocular lens (IOL) selection, and intraoperative aberrometry may help provide more predictable refractive outcomes.

In general, high myopes are at increased risk for retinal detachment following cataract surgery and should undergo preoperative dilated examination of the retinal periphery to assess for lattice degeneration and/or breaks [45]. Intraoperatively, maintaining the anterior chamber via careful wound construction to avoid leaks and using viscoelastic and irrigation to avoid sudden decompression of the eye are essential to mitigate the risk of vitreous movement. Additionally, a lower bottle height and shorter clear corneal wound facilitate operating in a deeper anterior chamber. Use of a chopping technique for nuclear disassembly may be preferable over a divide-and-conquer technique, both to enable operating in a deep chamber and to reduce stress on the zonules, which are known to be weaker in highly myopic eyes [43]. Surgeons should be alert to sudden posterior iris bowing, anterior chamber deepening, and pupillary dilation, which are typical of lens–iris diaphragm retropulsion syndrome [46]. This phenomenon, common in highly myopic and/or vitrectomized eyes, results from reverse pupillary block and can be resolved by gently separating the iris from the anterior capsule using a second instrument to permit equilibration of pressure in the anterior and posterior chambers. Thorough irrigation and aspiration to remove viscoelastic and residual lens particles from a deeper ciliary sulcus space in a myopic eye is important to prevent IOP elevation in the immediate postoperative period. Anterior capsular polishing is advisable to lower the risk of anterior capsular contraction syndrome, which is more common in myopic eyes and can lead to IOL subluxation [47,48].

Laser keratorefractive surgery is commonly performed in myopic eyes, and patients should be counseled preoperatively regarding the association between myopia and increased risk of glaucoma. Obtaining preoperative baseline glaucoma testing, including perimetry, OCT RNFL, and disc photography, is important in refractive surgery candidates who are deemed glaucoma suspects based on optic disc morphology and/or family history. Flap construction during laser-assisted in situ keratomileusis (LASIK), whether using a femtosecond laser or microkeratome, is associated with acute, severe IOP elevation that can further compromise an optic nerve with existing glaucomatous damage [49]. The newer small incision lenticule extraction (SMILE) procedure may offer an improved safety profile over LASIK in myopic glaucoma suspects given its flapless technique and generally limited postoperative steroid course. While photorefractive keratectomy (PRK) also avoids creation of a flap and concomitant IOP spikes, prolonged postoperative treatment with topical steroids required after PRK may result in IOP elevation in susceptible eyes. Interface fluid syndrome (IFS) is a rare postoperative complication that may develop after LASIK, and less commonly SMILE, in which high IOP (often, but not always, resulting from steroid use) causes an accumulation of fluid within the interface between the flap and the stroma [50,51,52]. Patients typically present with decreased vision and corneal haze, with or without visible fluid layering within the interface. A high degree of vigilance is required to detect IFS as IOP readings may be artificially low, especially when measured using a Goldmann applanation tonometer. Management of IFS consists of discontinuing steroids and initiating IOP-lowering medications, and incisional glaucoma surgery may be required in refractory cases [51]. The presence of an interface cyst or loose flap after LASIK or SMILE can also result in falsely low IOP readings. In general, measuring the IOP using several different methods, such as dynamic contour tonometry or pneumotonometry, is recommended in eyes with prior keratorefractive surgery given its effects on corneal biomechanics.

## 5. Conclusions

As myopia prevalence increases worldwide, our understanding of its complex pathophysiologic relationship to glaucoma must improve to lessen the global burden of functional glaucomatous vision loss in myopic eyes. Currently available imaging modalities of the peripapillary RNFL and macula are limited in their glaucoma diagnostic utility in myopic eyes due to nonrepresentative normative databases and artifacts resulting from characteristic anatomic features of myopic eyes. Visual field defects that are typical in glaucoma may also be observed in myopic eyes without glaucoma, further complicating its diagnosis. Given these diagnostic challenges, longitudinal follow-up of myopic eyes using perimetry, OCT RNFL, macular imaging, and disc photography is imperative to identify conversion to, or progression of, glaucoma. Surgical options for myopic eyes with glaucoma have expanded to include newer angle-based MIGS procedures, which may mitigate some of the vision-threatening complications more commonly observed with traditional glaucoma surgery, albeit with less efficacy. Myopic eyes are at increased risk of specific intraoperative and postoperative complications associated with cataract surgery, requiring careful surgical planning and modifications to standard phacoemulsification techniques. Laser keratorefractive surgery is associated with a risk of intraoperative and postoperative IOP elevation as well as unique complications that require a high degree of vigilance to detect. Additional research and developments in ocular imaging and perimetry will enhance our ability to more accurately detect glaucoma in myopic eyes, while advances in surgical innovation will improve the safety profile of glaucoma operations and help preserve vision in myopic patients.

## 6. Future Directions

Myopia represents a significant public health burden with the potential for far-reaching clinical, economic, and societal impacts. Curbing the myopia epidemic will depend upon the identification of risk factors and the development and implementation of strategies to prevent myopia onset or detect its presence early and slow its progression. Various risk factors for myopia development and progression have been studied in recent years, including environmental contributors such as urbanization [53] and decreased outdoor time [54]. Targeted interventions include increasing outdoor time during childhood. While robust evidence exists supporting the favorable effect of outdoor time on delaying or preventing myopia onset, published studies offer mixed results regarding its impact on reducing myopia progression [55]. Orthokeratology appears to correct low-to-moderate myopia and slow its progression via corneal reshaping, but concerns exist regarding its associated risk of infectious keratitis [56]. Low-dose atropine offers improved tolerability and less rebound myopic progression following its discontinuation compared to higher doses, but at the expense of decreased treatment effect [57]. Additional studies of myopia prevention and control strategies in diverse patient populations and geographic regions with long-term follow-up are needed to identify safe and effective interventions that can be implemented on a global scale. 

New strategies will also be necessary to improve early diagnosis of myopia as well as of glaucoma in myopic eyes, given the constraints of existing diagnostic techniques. Artificial intelligence, machine learning, and deep learning have the potential to revolutionize the future of myopia detection and prediction of disease progression. In addition, these strategies may allow for early identification of potentially vision-threatening sequelae of myopia, including glaucoma. Early work shows promise in using fundus photographs to train artificial intelligence platforms to detect glaucoma in populations with a high prevalence of myopia [58]. Using other existing imaging modalities, including OCT and visual fields, to train and validate artificial intelligence algorithms would transform our ability to diagnose and predict glaucomatous progression, which is especially important in complex clinical scenarios such as co-existing myopia and glaucoma. However, several limitations to the widespread implementation of artificial intelligence currently exist. These include reliance on a large number of high-quality images, the expense of imaging equipment, and medicolegal considerations. Moreover, validation of artificial intelligence algorithms would require data sharing among institutions, which may be limited by concerns regarding patient privacy and Health Insurance Portability and Accountability Act (HIPAA) compliance. Surmounting these and other as-yet-unforeseen challenges relating to artificial intelligence will require collaboration among clinicians, scientists, and regulatory agencies.

## Figures and Tables

**Table 1:** Advantages and disadvantages of glaucoma surgical procedures in myopic eyes.

Glaucoma Surgical Procedure	Advantages	Disadvantages	Special Considerations in Myopic Eyes
Microinvasive glaucoma surgery (MIGS)	Improved safety profile	Angle-based MIGS offer lower efficacy and may not be appropriate for patients with advanced glaucoma Hypotony may occur with bleb-based MIGS	Perform preoperative gonioscopy to assess angle anatomy in candidates for angle-based MIGS Use antimetabolites judiciously if performing bleb-based MIGS to avoid hypotony
Tube shunt surgery	Improved efficacy	Risk of hypotony and hypotony-related complications	Exercise caution while securing endplate to thin sclera Consider using valved or smaller surface area nonvalved implant Consider performing planned laser tube ligature release if using nonvalved implant
Trabeculectomy	Improved efficacy	Risk of hypotony and hypotony-related complications	Exercise caution during scleral flap dissection as sclera may be thin Use an increased number of, and tighter, scleral flap sutures to avoid hypotony Use antimetabolites judiciously to avoid hypotony
Trans-scleral cyclophotocoagulation	Nonincisional	Risk of hypotony and hypotony-related complications	Consider alternative to retrobulbar or peribulbar block to avoid risk of scleral perforation Consider scleral transillumination to confirm ciliary body location

## Data Availability

The data used in this review article are derived from published studies and are publicly available.
